# Synthesis and crystal structure of (2*S*,4a*R*,8a*R*)-6-oxo-2,4a,6,8a-tetra­hydro­pyrano[3,2-*b*]pyran-2-carboxamide

**DOI:** 10.1107/S2056989020001292

**Published:** 2020-04-30

**Authors:** John Greene, Noa Kopplin, Jack Roireau, Mark Bezpalko, Scott Kassel, Michael W. Giuliano, Robert Giuliano

**Affiliations:** a Department of Chemistry, Villanova University, 800 E Lancaster Avenue, Villanova, PA, USA; b Department of Chemistry and Biochemistry, College of Charleston, 66 George Street, Charleston, SC, USA

**Keywords:** diplopyrone, pyran­opyran, crystal structure

## Abstract

The pyran­opyran amide (2*S*,4a*R*,8a*R*)-6-oxo-2,4a,6,8a-tetra­hydro­pyrano[3,2-*b*]pyran-2-carboxamide, C_9_H_9_NO_4_, **3**, was prepared by a chemoselective hydration of the corresponding nitrile, **2**, using a heterogeneous catalytic method based on copper(II) supported on mol­ecular sieves, in the presence of acetaldoxime. Compound **3** belongs to a new class of pyran­opyrans that possess anti­bacterial and phytotoxic activity. Crystallographic analysis of **3** shows a bent structure for the *cis*-fused bicyclic pyran­opyran, similar to nitrile **2**. Evidence of an intra­molecular hydrogen bond involving the amide group and ring oxygen was not observed; however, two separate inter­molecular hydrogen-bonding inter­actions were observed between the amide hydrogen atoms and adjacent carbonyl oxygen atoms along the *b*- and *a*-axis directions. The latter inter­action may also be supported by an inter­molecular C—H⋯O hydrogen bond. The lattice is filled out by close-packed layers of this hydrogen-bonded network along the *c-*axis direction, related from one to the next by a 2_1_ screw axis.

## Abstract 2

(2*S*,4a*R*,8a*R*)-6-Oxo-2,4a,6,8a-tetra­hydro­pyrano[3,2-*b*]pyran-2-carboxamide, a potentially effective anti­bacterial agent, was prepared by a chemoselective hydration of the corresponding nitrile using a heterogeneous catalytic method based on copper(II) supported on mol­ecular sieves, in the presence of acetaldoxime. It belongs to a new class of pyran­opyrans that possess anti­bacterial and phytotoxic activity.

## Chemical context   

The phytotoxin diplopyrone **1** was isolated from the fungus *Diplodia mutila* and reported in 2003 (Evidente *et al.*, 2003 ▸). This fungus is considered a causative agent of cork oak decline and diplopyrone is implicated as the main phytotoxin responsible for this disease, the economic and environmental impacts of which are well known (Giorgio *et al.*, 2005 ▸). The proposed structure of diplopyrone contains a *cis*-fused pyran­opyran core and four chirality centers, originally assigned as 9*S*,6*R*,8a*S*,4a*S*, but revised recently to 9*R*,6*S*,8a*S*,4a*S* (Fusè *et al.*, 2019 ▸). In 2019, our laboratory published the synthesis and biological evaluation of pyran­opyran analogs based on the structure of diplopyrone (Lazzara *et al.*, 2019 ▸). These enanti­omeric analogs showed anti­bacterial and phytotoxic activity, in one case exceeding the activity of a commercially used anti­biotic that is used to treat bacterial diseases in pond-raised catfish, which is the largest segment of aqua­culture in the United States. Pyran­opyran nitrile **2** was approximately 100 times more potent in bioassay than florfenicol against *Edwardsiella ictaluri*, which causes enteric septicemia (ESC), a disease that can result in losses of tens of millions of dollars to the industry annually. Compound **2** was also phytotoxic in an assay using the aqua­tic plant *Lemna paucicostata* (L.) Hegelm. (duckweed). As part of our ongoing efforts to synthesize additional analogs of **1** for testing as new anti­bacterials and herbicides, we have recently prepared amide **3**, by a heterogeneous catalytic method that uses copper(II) supported on mol­ecular sieves, in the presence of acetaldoxime to carry out chemoselective hydration of **2** (Kiss & Hell, 2011 ▸).
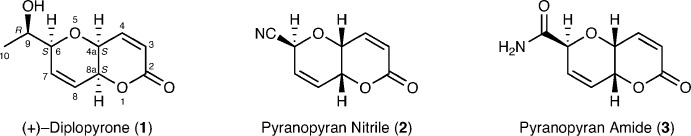


## Structural commentary   

**Figure 1:**
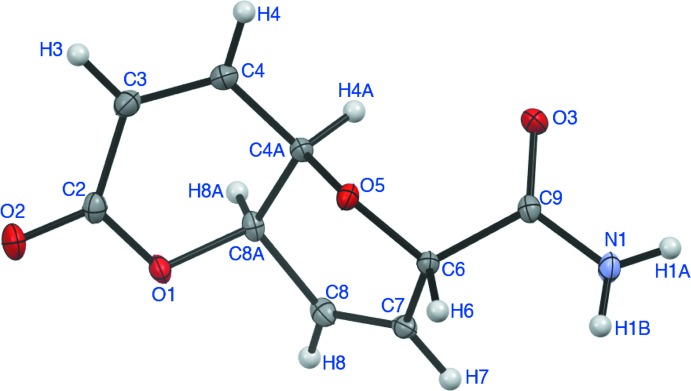
Mol­ecular structure of **3** with displacement ellipsoids at the 50% probability level.

Pyran­opyrans in which the two rings are *cis*-fused are relatively rare compared to *trans*-fused pyran­opyrans (Giuliano, 2014 ▸). A consequence of the *cis* ring fusion is that the mol­ecule has more of a bent shape than it would if *trans*-fused, which is demonstrated by the O1—C8*A*—C4*A*—O5 torsion angle of 72.95 (15)° *versus* 177° for a comparable *trans*-fused pyran­opyran (Yu *et al.*, 2017 ▸). Both rings adopt half-chair conformations, placing the amide group in a near 1,3-diaxial inter­action with H4*A*. These features are consistent with the results in the computational study reported (Evidente *et al.*, 2003 ▸). The study suggests the hy­droxy­ethyl side chain is involved in an intra­molecular hydrogen bond between the hydroxyl group and the O5 ring oxygen. By contrast, the amide side chain in **3** does not exhibit a similar intra­molecular hydrogen bond with its amino group in the solid state, as shown in Fig. 1 ▸. The overall structure of **3** is nearly identical to that of the pyran­opyran nitrile **2** with obvious deviation at the side chain.

The NMR spectra of the pyran­opyran amide **3** are similar to those of pyran­opyran nitrile **2**. The most obvious difference in the ^13^C spectra is the presence of the additional (amide) carbonyl carbon in **3** at δ 174.1 ppm and the absence of the nitrile carbon that occurs at δ 114.9 in **2**. The ^1^H spectrum of **3** shows slight changes in the chemical shifts of most protons, for example there is a downfield shift of H4*A* from δ 4.45 ppm in the nitrile to δ 4.61 ppm in the amide. The vinyl hydrogen H4 is also further downfield in the amide (δ 7.10 ppm *vs* 6.91 ppm). The torsion angle of 45.8° for H4*A*—C4*A*—C8*A*—H8*A* in **3** is consistent with the observed vicinal coupling constant of 4.5 Hz for H4*A*—H8*A* in the associated ^1^H NMR spectrum.

## Supra­molecular features   

**Figure 2:**
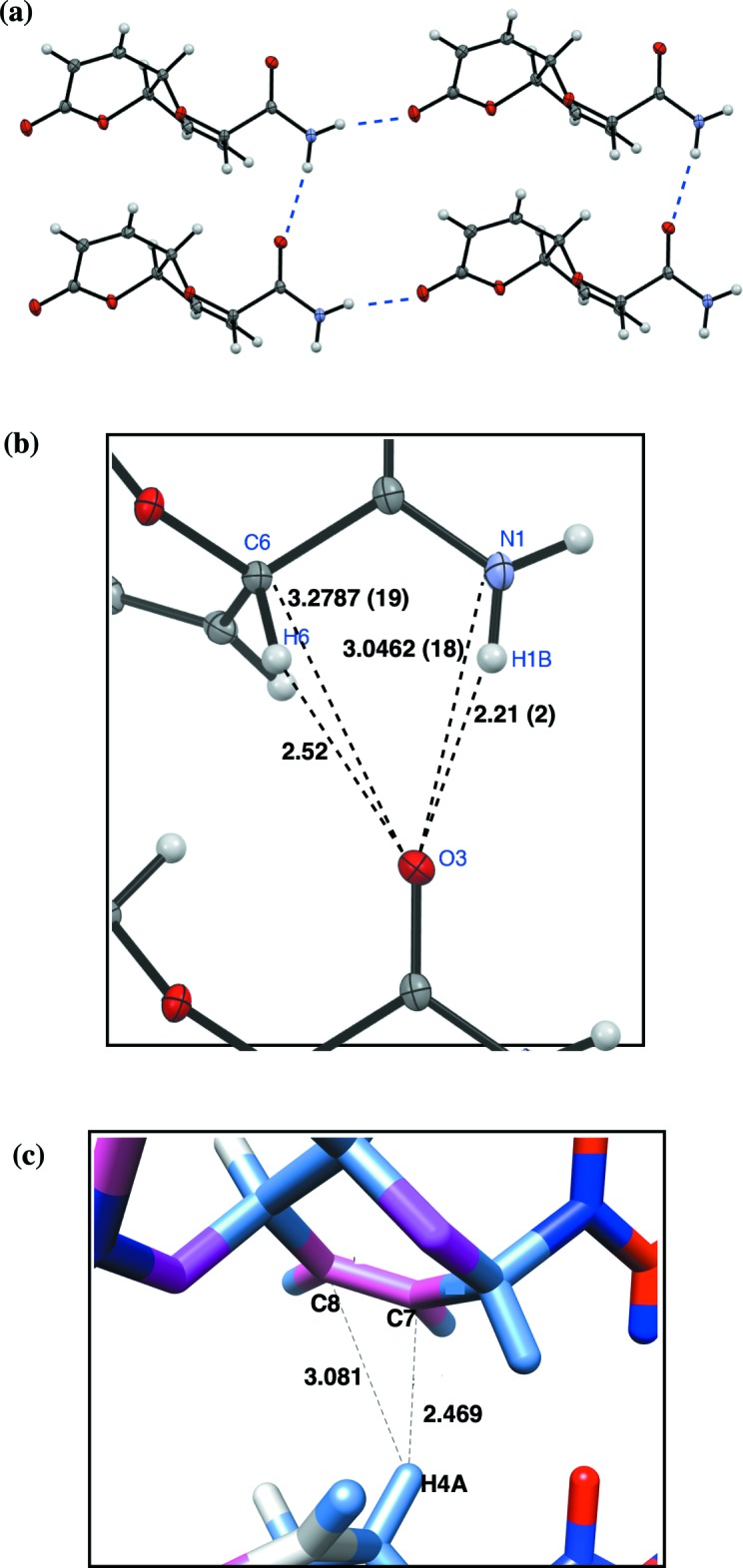
(*a*) Hydrogen-bonding inter­actions between copies of **3** along the *a* and *b* axes. (*b*) C—O and H—O distances suggestive of a potential C—H⋯O hydrogen bond along the *a* axis. N—H⋯O distances for the H1*B*⋯O3 hydrogen bond are included for comparison. (*c*) Measured distances and electrostatic coloring between H4*A*, C7, and C8 used to explore a potential C—H⋯π inter­action within the *ab* plane mol­ecular layers. Partial charges were generated within UCSF Chimera (Pettersen *et al.*, 2004 ▸) using the Amber ff14SB forcefield in Antechamber (Wang *et al.*, 2006 ▸) with the semi-empirical AM1 − BCC method and color coded with pink for negative charges and light blue for positive charges.

The amino hydrogen atoms of **3** are involved in inter­molecular hydrogen bonding with adjacent carbonyl oxygen atoms: H1*A* with O2^i^ and H1*B* with O3^ii^ (Fig. 3, Table 1 ▸, Symmetry codes: (i) *x*, *y* + 1, *z*; (ii) *x* + 1, *y*, *z*.). A packing diagram of **3** (Fig. 2 ▸ *a*) shows the N—H⋯O hydrogen-bonding inter­actions forming mol­ecular planes defined by the crystallographic *a-* and *b*-axes; packing of these hydrogen-bonded layers appears to be a function of solvent exclusion and van der Waals contact alone, lacking any hydrogen bonding.

The hydrogen-bonded network in the *ab* plane also presents an arrangement of C—H⋯O and C—H⋯π inter­actions that suggests two potential additional forces at play within the lattice of **3**. Fig. 2 ▸ *b* and Table 1 ▸ depict distances between H6 and O3^ii^ and C6 and O3^ii^ of adjacent copies of **3**. These distances fall within parameters for C—H⋯O hydrogen bonding as has been described in well-characterized membrane proteins and peptidomimetics (Senes *et al.*, 2001 ▸; Giuliano *et al.*, 2009 ▸); the α-protons implicated in these systems are structurally analogous to the C6—H6 bond of **3**. While we will not speculate on the energetic significance of this inter­action, which can arise as a coincidence of crystal packing (Dunitz & Gavezzotti, 2005 ▸), we note that such inter­actions have been spectroscopically measured within the core of the dimeric membrane peptide glycophorin A (Arbely & Arkin, 2004 ▸). Further, solid-state NMR studies have observed that ^1^H and ^13^C NMR shifts change for anomeric C—H bonds in crystalline maltose samples, suggesting that such interactions as described in this study (the C6—H6 bond in **3** is pseudo-anomeric) are not consequences of an energet­ically dominant lattice arrangement and N—H⋯O hydrogen bonding, but rather have some measurable, albeit weak, energetic contribution to inter­molecular association (Yates *et al.*, 2005 ▸).

Within the *ab* plane, H4*A* of one copy of **3** comes into close approach with its closest neighbor along the *a* axis. Fig. 3*c* depicts these distances, which place the centroid of the C7=C8 double bond within distance parameters similar to those calculated for aliphatic C—H⋯π inter­actions (Karthikeyan *et al.*, 2013 ▸). We investigated this further using a semi-empirical protocol to generate partial charges for the atoms of **3**. This only allows for qualitative comparison, and, as the color coding in Fig. 2 ▸ *c* reveals, the C7=C8 bond (pink, negative) is electrostatically matched with H4*A* (light blue, positive). Proper exploration of this would require more advanced QM/MM methods, however, the crystal packing of **3** is at least suggestive of a favorable geometry and electrostatic environment for C—H⋯π inter­actions.

## Database survey   

A search of the Cambridge Structural Database (CSD Version 5.41, November 2019; Groom *et al.*, 2016 ▸) using the core fused ring lactone in the search query revealed only three similar structures (Somarathne *et al.*, 2019 ▸; Lazzara *et al.*, 2019 ▸) in which the pyran­opyran ring system is *cis*-fused and the two double bonds are in the same location as they are in **3**. Among the total 40 structures that were found in the search, the pyran­opyran core of several were *trans*-fused, for example, the bergenins and also truncated ladder ethers related to brevitoxin. Some compounds possessed aryl rings fused to the pyran­opyran system while others had double bonds at alternate positions including the ring junction.

## Synthesis and crystallization   

**(2** ***S*** **,4a** ***R*** **,8a** ***R*** **)-6-Oxo-2,4a,6,8a-tetra­hydro­pyrano[3,2-** ***b*** **]pyran-2-carboxamide 3:**

Compound **3** was prepared by the procedure of Kiss & Hell (2011 ▸) with a change of solvent from methanol to *tert*-butanol. A mixture of (4a*R*,6*S*,8a*R*)-6-cyano-6,8a-di­hydro­pyrano-[3,2-*b*]pyran-2-(4a*H*)-one **2** (0.040 g, 0.226 mmol), Cu^II^-4 Å catalyst (0.022 g), acetaldoxime (0.040 g, 0.678 mmol) and *tert*-butanol (2 mL) was stirred at 343 K for 4 h. The mixture was filtered through a pad of Celite and concentrated to a yellow–brown solid that was purified by cartridge chromatography on a Waters vacuum manifold system using 5% methanol/chloro­form as eluant (flash chromatography was also successful using 10% methanol/chloro­form). Concentration of fractions left a white solid; yield, 0.0227 g (51.5%). Single crystals were obtained from a solution of **3** in 10% methanol/chloro­form at 253 K. *R_f_* = 0.2 (10% methanol/ chloro­form); mp 433-437 K; [α]_D_^20^ −268 (*c*, 0.8, methanol); IR (ATR) ν 3425, 3325, 3219, 1710, 1670, 1618 cm^−1^; ^1^H NMR (300 MHz, CDCl_3_) δ 7.10 (*dd*, 1H, *J*_3,4_ = 10.1, *J*_4,4a_ = 5.4 Hz, H-4), 6.40 (*ddd*, 1H, *J*_7,8_ = 10.2, *J*_6,7_ = 3.6, *J*_7,8a_ = 1.2 Hz, H-7), 6.16 (*d*, 1H, *J*_3,4_ = 10.5, H-3), 6.10 (*m*, 1H, H-8), 4.86 (*bs*, 2H, NH_2_), 4.80 (*m*, 2H, H-6, H-8a), 4.61 (*ddd*, 1H, *J*_4a,4_ = *J*_4a,8a_ = 4.5, *J*_4a,8_ = 1.2 Hz, H-4a); ^13^C{^1^H} NMR (CDCl_3_) δ 174.1, 164.8, 143.7, 131.8, 124.5, 123.3, 74.0, 70.2, 64.4. HRMS (ESI–TOF) *m*/*z* calculated for C_9_H_10_NO_4_ 196.0610, found 196.0607.

## Refinement   

Crystal data, data collection, and structure refinement details are summarized in Table 2 ▸. The absolute configuration was known from the synthetic route and assigned accordingly. The amino hydrogen atoms were found in the electron difference map and refined isotropically, while all other hydrogen atoms were treated as idealized contributions with C—H = 0.95–1.00 Å and *U*_iso_(H) = 1.2*U*_eq_(C).

## Supplementary Material

Crystal structure: contains datablock(s) I. DOI: 10.1107/S2056989020001292/zl4038sup1.cif

Structure factors: contains datablock(s) I. DOI: 10.1107/S2056989020001292/zl4038Isup2.hkl

Supporting information file. DOI: 10.1107/S2056989020001292/zl4038Isup3.cml

CCDC reference: 1980773

Additional supporting information:  crystallographic information; 3D view; checkCIF report

## Figures and Tables

**Table 1:** Hydrogen-bond geometry (Å, °)

*D*—H⋯*A*	*D*—H	H⋯*A*	*D*⋯*A*	*D*—H⋯*A*
N1—H1*A*⋯O2^i^	0.86 (3)	2.08 (3)	2.8840 (18)	156 (2)
N1—H1*B*⋯O3^ii^	0.87 (2)	2.21 (2)	3.0462 (18)	163.1 (19)
C6—H6⋯O3^ii^	1.00	2.52	3.2787 (19)	133

Symmetry codes: (i) 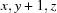; (ii) 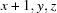.

**Table 2:** Experimental details

Crystal data	
Chemical formula	C_9_H_9_NO_4_
*M*_r_	195.17
Crystal system, space group	Orthorhombic, *P*2_1_2_1_2_1_
Temperature (K)	100
*a*, *b*, *c* (Å)	4.9279 (1), 10.6350 (3), 15.8788 (4)
*V* (Å^3^)	832.18 (4)
*Z*	4
Radiation type	Mo *K*α
μ (mm^−1^)	0.12
Crystal size (mm)	0.4 × 0.3 × 0.18

Data collection	
Diffractometer	Bruker SMART APEXII area detector
Absorption correction	Multi-scan (*SADABS*; Krause *et al.*, 2015 ▸)
*T*_min_, *T*_max_	0.654, 0.746
No. of measured, independent and observed [*I* > 2σ(*I*)] reflections	14252, 2461, 2366
*R*_int_	0.031
(sin θ/λ)_max_ (Å^−1^)	0.708

Refinement	
*R*[*F* ^2^ > 2σ(*F* ^2^)], *wR*(*F* ^2^), *S*	0.032, 0.080, 1.04
No. of reflections	2461
No. of parameters	135
H-atom treatment	H atoms treated by a mixture of independent and constrained refinement
Δρ_max_, Δρ_min_ (e Å^−3^)	0.35, −0.20
Absolute structure	Flack *x* determined using 928 quotients [(*I* ^+^)−(*I* ^−^)]/[(*I* ^+^)+(*I* ^−^)] (Parsons et al., 2013 ▸)
Absolute structure parameter	−0.1 (3)

Computer programs: *APEX3* (Bruker, 2016 ▸), *SAINT* (Bruker, 2015 ▸), *SIR92* (Altomare *et al.*, 1994 ▸), *SHELXL2014* (Sheldrick, 2015 ▸) and *OLEX2* (Dolomanov *et al.*, 2009 ▸).

## supplementary crystallographic information

### Crystal data

**Table:**

C_9_H_9_NO_4_	*D*_x_ = 1.558 Mg m^−^^3^
*M**_r_* = 195.17	Mo *K*α radiation, λ = 0.71073 Å
Orthorhombic, *P*2_1_2_1_2_1_	Cell parameters from 8408 reflections
*a* = 4.9279 (1) Å	θ = 2.3–30.2°
*b* = 10.6350 (3) Å	µ = 0.12 mm^−^^1^
*c* = 15.8788 (4) Å	*T* = 100 K
*V* = 832.18 (4) Å^3^	Prism, colourless
*Z* = 4	0.4 × 0.3 × 0.18 mm
*F*(000) = 408	

### Data collection

**Table:**

Bruker SMART APEXII area detector diffractometer	2461 independent reflections
Radiation source: sealed tube	2366 reflections with *I* > 2σ(*I*)
Graphite monochromator	*R*_int_ = 0.031
Detector resolution: 8 pixels mm^-1^	θ_max_ = 30.2°, θ_min_ = 2.3°
ω and φ scans	*h* = −5→6
Absorption correction: multi-scan (SADABS; Krause *et al*., 2015)	*k* = −15→14
*T*_min_ = 0.654, *T*_max_ = 0.746	*l* = −22→22
14252 measured reflections	

### Refinement

**Table:**

Refinement on *F*^2^	Hydrogen site location: mixed
Least-squares matrix: full	H atoms treated by a mixture of independent and constrained refinement
*R*[*F*^2^ > 2σ(*F*^2^)] = 0.032	*w* = 1/[σ^2^(*F*_o_^2^) + (0.0446*P*)^2^ + 0.205*P*] where *P* = (*F*_o_^2^ + 2*F*_c_^2^)/3
*wR*(*F*^2^) = 0.080	(Δ/σ)_max_ < 0.001
*S* = 1.04	Δρ_max_ = 0.35 e Å^−^^3^
2461 reflections	Δρ_min_ = −0.20 e Å^−^^3^
135 parameters	Absolute structure: Flack *x* determined using 928 quotients [(*I*^+^)-(*I*^-^)]/[(*I*^+^)+(*I*^-^)] (Parsons et al., 2013)
0 restraints	Absolute structure parameter: −0.1 (3)
Primary atom site location: structure-invariant direct methods	

### Special details

**Table:**

Geometry. All esds (except the esd in the dihedral angle between two l.s. planes) are estimated using the full covariance matrix. The cell esds are taken into account individually in the estimation of esds in distances, angles and torsion angles; correlations between esds in cell parameters are only used when they are defined by crystal symmetry. An approximate (isotropic) treatment of cell esds is used for estimating esds involving l.s. planes.
Refinement. 1. Fixed Uiso At 1.2 times of: All C(H) groups 2.a Ternary CH refined with riding coordinates: C4A(H4A), C6(H6), C8A(H8A) 2.b Aromatic/amide H refined with riding coordinates: C3(H3), C4(H4), C7(H7), C8(H8) 3. N1(H1A) and N1(H1B) located from the difference map with refined Uiso

### Fractional atomic coordinates and isotropic or equivalent isotropic displacement parameters (Å^2^)

**Table:**

	*x*	*y*	*z*	*U*_iso_*/*U*_eq_	
O1	0.7129 (2)	0.30730 (10)	0.31022 (7)	0.0145 (2)	
C2	0.5987 (3)	0.22091 (15)	0.36070 (9)	0.0141 (3)	
O2	0.7069 (3)	0.11890 (11)	0.36663 (8)	0.0198 (3)	
C3	0.3563 (3)	0.25659 (15)	0.40997 (9)	0.0155 (3)	
H3	0.2482	0.1928	0.4350	0.019*	
C4	0.2869 (3)	0.37655 (14)	0.41980 (10)	0.0154 (3)	
H4	0.1413	0.3982	0.4562	0.019*	
C4A	0.4377 (3)	0.47756 (14)	0.37369 (9)	0.0121 (3)	
H4A	0.3071	0.5453	0.3574	0.015*	
O5	0.6340 (2)	0.52880 (10)	0.43085 (6)	0.0118 (2)	
C6	0.7796 (3)	0.63226 (13)	0.39613 (9)	0.0107 (3)	
H6	0.9519	0.6405	0.4289	0.013*	
C7	0.8579 (3)	0.61248 (14)	0.30510 (9)	0.0126 (3)	
H7	0.9754	0.6718	0.2792	0.015*	
C8	0.7700 (3)	0.51613 (14)	0.25988 (9)	0.0140 (3)	
H8	0.8362	0.5046	0.2042	0.017*	
C8A	0.5687 (3)	0.42536 (14)	0.29495 (9)	0.0124 (3)	
H8A	0.4244	0.4103	0.2518	0.015*	
C9	0.6280 (3)	0.75809 (14)	0.40406 (8)	0.0112 (3)	
O3	0.3793 (2)	0.76516 (11)	0.40436 (7)	0.0163 (2)	
N1	0.7940 (3)	0.85729 (13)	0.40610 (9)	0.0151 (3)	
H1A	0.735 (5)	0.933 (2)	0.4068 (15)	0.033 (6)*	
H1B	0.967 (5)	0.845 (2)	0.4125 (13)	0.017 (5)*	

### Atomic displacement parameters (Å^2^)

**Table:**

	*U*^11^	*U*^22^	*U*^33^	*U*^12^	*U*^13^	*U*^23^
O1	0.0173 (5)	0.0092 (5)	0.0170 (5)	0.0032 (4)	0.0028 (4)	−0.0007 (4)
C2	0.0161 (7)	0.0111 (7)	0.0151 (6)	−0.0009 (5)	−0.0026 (5)	−0.0014 (5)
O2	0.0235 (6)	0.0102 (5)	0.0255 (6)	0.0034 (4)	0.0003 (5)	0.0013 (4)
C3	0.0137 (6)	0.0138 (7)	0.0189 (7)	−0.0026 (6)	0.0003 (5)	0.0016 (6)
C4	0.0121 (6)	0.0140 (7)	0.0202 (7)	−0.0014 (5)	0.0027 (5)	−0.0012 (5)
C4A	0.0104 (6)	0.0093 (6)	0.0166 (6)	0.0006 (5)	0.0004 (5)	−0.0011 (5)
O5	0.0140 (5)	0.0093 (5)	0.0121 (4)	−0.0021 (4)	0.0006 (4)	0.0006 (4)
C6	0.0103 (6)	0.0087 (6)	0.0130 (6)	−0.0001 (5)	−0.0001 (5)	0.0005 (5)
C7	0.0116 (6)	0.0119 (6)	0.0145 (6)	0.0021 (5)	0.0030 (5)	0.0029 (5)
C8	0.0170 (7)	0.0133 (7)	0.0117 (6)	0.0035 (6)	0.0023 (5)	0.0018 (5)
C8A	0.0153 (7)	0.0088 (6)	0.0130 (6)	0.0021 (5)	−0.0017 (5)	−0.0005 (5)
C9	0.0144 (6)	0.0096 (6)	0.0097 (5)	0.0009 (5)	0.0001 (5)	−0.0001 (5)
O3	0.0121 (5)	0.0128 (5)	0.0240 (5)	0.0019 (4)	0.0016 (4)	−0.0016 (4)
N1	0.0155 (6)	0.0082 (6)	0.0215 (6)	0.0000 (5)	−0.0023 (5)	0.0015 (5)

### Geometric parameters (Å, º)

**Table:**

O1—C2	1.3428 (18)	C6—H6	1.0000
O1—C8A	1.4629 (18)	C6—C7	1.511 (2)
C2—O2	1.2126 (19)	C6—C9	1.538 (2)
C2—C3	1.478 (2)	C7—H7	0.9500
C3—H3	0.9500	C7—C8	1.324 (2)
C3—C4	1.330 (2)	C8—H8	0.9500
C4—H4	0.9500	C8—C8A	1.492 (2)
C4—C4A	1.497 (2)	C8A—H8A	1.0000
C4A—H4A	1.0000	C9—O3	1.2280 (19)
C4A—O5	1.4343 (18)	C9—N1	1.335 (2)
C4A—C8A	1.513 (2)	N1—H1A	0.86 (3)
O5—C6	1.4243 (17)	N1—H1B	0.87 (2)
			
C2—O1—C8A	118.85 (12)	C7—C6—C9	108.88 (11)
O1—C2—C3	118.63 (14)	C9—C6—H6	107.1
O2—C2—O1	118.31 (14)	C6—C7—H7	118.6
O2—C2—C3	122.97 (14)	C8—C7—C6	122.88 (13)
C2—C3—H3	119.5	C8—C7—H7	118.6
C4—C3—C2	121.08 (14)	C7—C8—H8	119.5
C4—C3—H3	119.5	C7—C8—C8A	121.06 (13)
C3—C4—H4	119.9	C8A—C8—H8	119.5
C3—C4—C4A	120.23 (14)	O1—C8A—C4A	112.66 (12)
C4A—C4—H4	119.9	O1—C8A—C8	107.10 (12)
C4—C4A—H4A	108.9	O1—C8A—H8A	108.7
C4—C4A—C8A	110.65 (12)	C4A—C8A—H8A	108.7
O5—C4A—C4	107.33 (12)	C8—C8A—C4A	110.78 (12)
O5—C4A—H4A	108.9	C8—C8A—H8A	108.7
O5—C4A—C8A	112.00 (12)	O3—C9—C6	122.57 (14)
C8A—C4A—H4A	108.9	O3—C9—N1	124.26 (15)
C6—O5—C4A	112.85 (11)	N1—C9—C6	113.09 (13)
O5—C6—H6	107.1	C9—N1—H1A	122.5 (17)
O5—C6—C7	113.04 (12)	C9—N1—H1B	118.8 (14)
O5—C6—C9	113.33 (12)	H1A—N1—H1B	118 (2)
C7—C6—H6	107.1		
			
O1—C2—C3—C4	−15.0 (2)	O5—C4A—C8A—C8	−47.00 (16)
C2—O1—C8A—C4A	41.32 (17)	O5—C6—C7—C8	8.0 (2)
C2—O1—C8A—C8	163.37 (12)	O5—C6—C9—O3	−30.03 (19)
C2—C3—C4—C4A	6.2 (2)	O5—C6—C9—N1	153.21 (13)
O2—C2—C3—C4	161.46 (16)	C6—C7—C8—C8A	4.7 (2)
C3—C4—C4A—O5	−98.05 (16)	C7—C6—C9—O3	96.70 (16)
C3—C4—C4A—C8A	24.4 (2)	C7—C6—C9—N1	−80.06 (15)
C4—C4A—O5—C6	−176.04 (11)	C7—C8—C8A—O1	−108.78 (16)
C4—C4A—C8A—O1	−46.75 (16)	C7—C8—C8A—C4A	14.4 (2)
C4—C4A—C8A—C8	−166.70 (12)	C8A—O1—C2—O2	173.12 (13)
C4A—O5—C6—C7	−41.21 (16)	C8A—O1—C2—C3	−10.21 (19)
C4A—O5—C6—C9	83.29 (14)	C8A—C4A—O5—C6	62.33 (15)
O5—C4A—C8A—O1	72.95 (15)	C9—C6—C7—C8	−118.85 (16)

### Hydrogen-bond geometry (Å, º)

**Table:**

*D*—H···*A*	*D*—H	H···*A*	*D*···*A*	*D*—H···*A*
N1—H1*A*···O2^i^	0.86 (3)	2.08 (3)	2.8840 (18)	156 (2)
N1—H1*B*···O3^ii^	0.87 (2)	2.21 (2)	3.0462 (18)	163.1 (19)
C6—H6···O3^ii^	1.00	2.52	3.2787 (19)	133

Symmetry codes: (i) *x*, *y*+1, *z*; (ii) *x*+1, *y*, *z*.
